# Z-drug abuse and dependence: clinical guideline of the Brazilian Academy of Neurology for diagnosis and management

**DOI:** 10.1055/s-0045-1812323

**Published:** 2025-11-04

**Authors:** Fernando Gustavo Stelzer, Andrea Bacelar, Alan Luiz Éckeli, André Brooking Negrão, Carlos Maurício Oliveira Almeida, Clélia Maria Ribeiro Franco, Gabriel Natan Pires, Lívia Leite Goés Gitaí, Manoel Alves Sobreira-Neto, Márcia Assis, Paulo Afonso Mei, Rosa Hasan, Sandra Cristina Gonçalves Martinez, Tania Marchiori, Thiago M. Fidalgo, Luciana L. de Siqueira, Dalva Poyares

**Affiliations:** 1 Universidade de São Paulo, Faculdade de Medicina de Ribeirão Preto, Departamento de Neurociências e Ciências do Comportamento, Ribeirão Preto SP, Brazil.; 2 Clínica Bacelar, Rio de Janeiro RJ, Brazil.; 3 Universidade de São Paulo, Faculdade de Medicina, Hospital das Clínicas, Instituto Perdizes, Departamento de Psiquiatra, São Paulo SP, Brazil.; 4 Universidade Estadual do Amazonas, Curso de Medicina, Disciplina de Neurologia e Neurocirurgia, Manaus AM, Brazil.; 5 Universidade Federal de Pernambuco, Serviço de Neurologia, Hospital das Clínicas, EBSERH. Recife PE, Brazil.; 6 Universidade Federal de São Paulo, Departamento de Psicobiologia, São Paulo SP, Brazil.; 7 Instituto do Sono, São Paulo SP, Brazil.; 8 Universidade Federal de Alagoas, Faculdade de Medicina, Maceió AL, Brazil.; 9 Centro Universitário CESMAC, Maceió AL, Brazil.; 10 Universidade Federal do Ceará, Faculdade de Medicina, Fortaleza CE, Brazil.; 11 Hospital São Lucas, Curitiba PR, Brazil.; 12 Universidade Federal de São Carlos, Departamento de Medicina, São Carlos SP, Brazil.; 13 Faculdade de Medicina de Jundiav, Departamento de Clvnica Médica, Jundiav, SP, Brazil.; 14 Universidade de São Paulo, Instituto de Psiquiatra, São Paulo SP, Brazil.; 15 Hospital da Restauração, Recife PE, Brazil.; 16 Universidade Estadual de Campinas, Faculdade de Ciências Médicas, Departamento de Neurologia, Campinas SP, Brazil.; 17 Universidade Federal de São Paulo, Escola Paulista de Medicina, Departamento de Psiquiatria, São Paulo SP, Brazil.

**Keywords:** Zolpidem, Eszopiclone, Substance-Related Disorders, Sleep Initiation and Maintenance Disorders

## Abstract

Benzodiazepine (BZD) receptor agonists, commonly known as Z-drugs, are non-BZD hypnotics primarily prescribed for the treatment of insomnia. Their use is recommended for no longer than four weeks to minimize the risk of adverse effects, including dependence and withdrawal. However, these guidelines are frequently disregarded, and the abuse of and dependence on Z-drugs has emerged as a growing public health concern in Brazil. The present article reviews the current evidence on Z-drug use disorder—including dependence and withdrawal—and proposes clinical guidelines for the management of discontinuation. The recommendations were developed based on a systematic review of the literature and refined using the Delphi methodology. The consensus was developed by a multidisciplinary task force, with coordination and voting led by a steering committee. An advisory committee, consisting of neurologists from the Brazilian Academy of Neurology (Academia Brasileira de Neurologia, ABN, in Portuguese) and psychiatrists specializing in substance-use disorders, contributed to the selection and organization of the scientific literature and took part in the voting process. Key recommendations were established: 1) prior to discontinuation, a comprehensive assessment of mental status, psychiatric and sleep comorbidities, and the degree of pharmacological dependence is essential; 2) gradual tapering is advised; 3) non-pharmacological interventions, such as cognitive behavioral therapy for insomnia, are *recommended* , and acceptance and commitment therapy, which is *optional* , may be incorporated; 4) for zolpidem withdrawal, adjunctive pharmacotherapy, which is *optional* , may include trazodone, other antidepressants, quetiapine or other antipsychotics, alpha-2-delta (α2δ) ligands, or alternative hypnotics (such as ramelteon, zopiclone, and eszopiclone); 5) for Z-drug discontinuation, intermediate- or long-acting BZDs are *recommended* ; and 6) short- or ultra-short-acting BZDs and immediate-release melatonin are *not recommended* .

## INTRODUCTION

Benzodiazepine (BZD) receptor agonists, or Z-drugs, were introduced to the market in the 1990s as safer sedative-hypnotic agents, with a lower potential for abuse and dependence compared to BZDs. 1 Although structurally distinct, both classes of medications (BZDs and Z-drugs) bind to the gamma-aminobutyric acid type-A (GABA _A_ ) receptor, increasing the frequency of opening of the chloride channel and, thereby, potentiating the inhibitory effect of GABA on the central nervous system (CNS). However, compared to BZDs, Z-drugs exhibit greater selectivity for specific α subunits of the GABA _A_ receptor, which might explain their reduced anxiolytic, muscle relaxant, and antiseizure effects. 1 2 3 The greater receptor selectivity of Z-drugs, combined with their short half-life, initially positioned them as ideal hypnotics, producing effects limited to sleep induction, with minimal or no residual effects the following day. 3

Z-drugs are currently indicated for the treatment of insomnia. Use of zolpidem and zopiclone beyond 4 weeks is not recommended due to the lack of long-term safety data. In contrast, eszopiclone has demonstrated safety for up to 12 months in primary insomnia patients. They are not recommended for pregnant or breastfeeding women, nor for children or adolescents. In older adults, the recommended doses are lower than those for younger individuals. 4 5 6

Zolpidem abuse was first reported in the early 1990s, 7 shortly after its introduction into the market in the United States (US) and Europe; the initial cases involved dose escalation due to tolerance and withdrawal symptoms, including seizures. Large numbers of intoxications were later described, 8 and additional reports 9 highlighted misuse motivated by psychomotor effects. These and subsequent publications underscored the abuse potential of Z-drugs, especially zolpidem.

In Brazil, zolpidem ranks among the most common drugs sold off the counter or illegally. Additionally, prescriptions for Z-drugs have risen significantly. In 2014, 338,367 boxes of zolpidem were sold, increasing to 810,353 in 2021; sales of zopiclone also rose during this period, from 15,060 boxes in 2014 to 83,910 in 2021. 10 Between 2014 and 2021, zolpidem was the 3rd most sold hypnotic in the country, accounting for 14.4% of total hypnotic sales, following clonazepam and alprazolam. 10 As of August 2024, the Brazilian Health Regulatory Agency (Agência Nacional de Vigilância Sanitária, ANVISA, in Portuguese) implemented tighter control on the prescription of the Z-drugs, given the irregular and abusive use – particularly of zolpidem. 11

With increased Z-drug use, a range of serious adverse effects has been identified, including delirium, sleep-related eating disorder, amnesia, and complex behaviors such as sleep-driving; these effects may occur at therapeutic doses, and they are intensified by alcohol or other sedatives. 6 12 13

The abuse of and dependence on Z-drugs has, therefore, emerged as a public health concern in Brazil. The present article aims to review the current evidence on Z-drug use disorder—including dependence and withdrawal—and to propose clinical guidelines for the management of discontinuation. In Brazil, three agents from this class are available: zolpidem, zopiclone, and eszopiclone, which are the focus of the current article.

## METHODS

### Composition of the task force

The present consensus was developed through the collaboration of authors with varying levels of participation. The task force consisted of experts, ensuring a comprehensive approach to the consensus. Each member contributed their unique insights, fostering a robust discussion that ultimately shaped the final recommendations. The steering committee, composed of authors AFRB, FGS, and GNP, was responsible for coordinating the process and participating in voting. The advisory committee included neurologists from the Brazilian Academy of Neurology (Academia Brasileira de Neurologia, ABN, in Portuguese; authors ALE, AP, CMOA, CMRF, DP, LLGG, MASN, MA, PAM, RH, SCGM, and TM), who contributed to the selection and organization of the scientific literature and took part in the voting. Psychiatrists specializing in substance use disorders (authors ABN, TMF) also participated in the voting process.

### Systematic reviews

Systematic reviews were conducted with two primary objectives: 1) to generate evidence for each of the research questions formulated; and 2) to provide a basis for the working groups to draft recommendations and guide discussions. The limited number of studies and high methodological heterogeneity prevented us from performing meta-analyses for any of the research questions. The methods used in the systematic reviews are described in the following subsections.

A total of 29 research questions were developed using the Population, Intervention, Comparison, Outcome (PICO) framework: 18 by the Z-drug deprescribing group, 4 by the Z-drug abstinence group, and 7 by the Z-drug dependence and abuse group ( Table 1 ). Systematic reviews were conducted for all questions, as detailed in **Supplementary Material** available at https://www.arquivosdeneuropsiquiatria.org/wp-content/uploads/2025/08/ANP-2025.0232-Supplementary-Material.pdf **Supplementary Material Figure S1** , with inclusion and exclusion criteria outlined in **Supplementary Material Table S1** .

**Table 1:** Research questions

PICO #	Question	Population	Intervention or exposure	Comparison	Outcome
**Z-drug deprescription group**					
1.1	What is the effect of CBT-I on Z-drug tapering in adults?	Adults with insomnia using Z-drugs	CBT-I	Placebo	Z-drug deprescription
1.2	What is the effect of CBT-I on Z-drug tapering in older adults?	Elderly with insomnia using Z-drugs	CBT-I	Placebo	Z-drug deprescription
1.3	What is the effect of pharmacological interventions, compared with placebo or non-pharmacological measures, on Z-drug tapering in adults?	Adults with insomnia using Z-drugs	Pharmacological treatment	Placebo or non-pharmacological measures (including CBT-I)	Z-drug deprescription
1.4	What is the effect of pharmacological interventions, compared with placebo or non-pharmacological measures, on Z-drug tapering in older adults?	Elderly individuals with insomnia using Z-drugs	Pharmacological treatment	Placebo or non-pharmacological measures (including CBT-I)	Z-drug deprescription
1.5	What is the effect of non-pharmacological interventions, compared with placebo or pharmacological treatment, on Z-drug tapering in adults?	Adults with insomnia using Z-drugs	Non-pharmacological treatment	Placebo and/or pharmacological treatment	Z-drug deprescription
1.6	What is the effect of non-pharmacological interventions, compared with placebo or pharmacological treatment, on Z-drug tapering in older adults?	Elderly individuals with insomnia using Z-drugs	Non-pharmacological treatment	Placebo and/or pharmacological treatment	Z-drug deprescription
1.7	What is the effect of inpatient versus outpatient treatment on Z-drug tapering or discontinuation in adults and older aldults?	Adults and elderly individuals	Hospital admission	Outpatient treatment	Z-drug deprescription
1.8	What is the effect of gradual tapering compared to abrupt discontinuation on Z-drug withdrawal outcomes?	Adults and elderly individuals	Gradual tapering	Abrupt discontinuation	Z-drug deprescription
1.9	What is the effect of clinical monitoring (in-person or remote) compared to no monitoring on the recurrence of Z-drug abuse?	Adults and elderly individuals	Regular clinical follow-up	No medical follow-up	Z-drug use relapse
1.10	What is the effect of antidepressant use, compared to gradual tapering or placebo, on Z-drug discontinuation in adults?	Adults with insomnia using Z-drugs	Pharmacological treatment	Placebo or gradual tapering	Z-drug use relapse
1.11	What is the effect of antidepressant use, compared to gradual tapering or placebo, on Z-drug discontinuation in older adults?	Elderly individuals with insomnia using Z-drugs	Pharmacological treatment	Placebo or gradual tapering	Z-drug use relapse
1.12	What is the effect of antipsychotic use, compared to gradual tapering or placebo, on Z-drug discontinuation in adults?	Adults with insomnia using Z-drugs	Pharmacological treatment	Placebo or gradual tapering	Z-drug use relapse
1.13	What is the effect of antipsychotic use, compared to gradual tapering or placebo, on Z-drug discontinuation in older adults?	Elderly individuals with insomnia using Z-drugs	Pharmacological treatment	Placebo or gradual tapering	Z-drug use relapse
1.14	What is the effect of benzodiazepine use, compared to gradual tapering or placebo, on Z-drug discontinuation in adults?	Adults with insomnia using Z-drugs	Pharmacological treatment	Placebo or gradual tapering	Z-drug use relapse
1.15	What is the effect of benzodiazepine use, compared to gradual tapering or placebo, on Z-drug discontinuation in older adults	Elderly individuals with insomnia using Z-drugs	Pharmacological treatment	Placebo or gradual tapering	Z-drug use relapse
1.16	When should hospitalization be recommended for adult patients undergoing zolpidem tapering?	Adults (aged 18–64 years)	Hospital admission	Outpatient treatment	Z-drug deprescription
1.17	When should hospitalization be recommended for older adult patients undergoing zolpidem tapering?	Elderly individuals (aged ≥ 65 years)	Hospital admission	Outpatient treatment	Z-drug deprescription
1.18	What is the recommended zolpidem tapering dose percentage and duration of treatment?	Adults and elderly individuals	Gradual tapering	Placebo or gradual tapering	Z-drug deprescription
**Z-drug withdrawal group**					
2.1	What symptoms are associated with Z-drug withdrawal?	Adults with insomnia using Z-drugs	Z-drug withdrawal	NA	Clinical manifestations
2.2	What is the prevalence of Z-drug withdrawal syndrome?	Adults with insomnia using Z-drugs	Z-drug withdrawal	NA	Prevalence
2.3	What is the recommended treatment for epileptic seizures associated with Z-drug withdrawal?	Adults with seizures due to Z-drug withdrawal	Any treatment	Any comparison	Epileptic seizures
2.4	What is the recommended treatment for rebound insomnia associated with Z-drug withdrawal?	Adults with rebound insomnia due to Z-drug withdrawal	Any treatment	Any comparison	Rebound insomnia
**Z-drug abuse and dependence group**					
3.1	What is the effect of a family history of substance dependence on the risk of Z-drug abuse?	General population	Family history of substance use disorder	No family history of substance use disorder	Z-drug abuse and dependence
3.2	What is the effect of a history of abuse of alcohol, sedatives, or other hypnotic drugs on the risk of Z-drug abuse?	General population	Previous substance use disorder or substance abuse disorder	No previous history of substance use disorder or substance abuse disorder	Z-drug abuse and dependence
3.3	What is the impact of a history of psychiatric illness (e.g., anxiety, depression, ADHD, personality disorders) on the risk of Z-drug abuse or the development of Z-drug use disorder?	General population	Previous mental disorder	No previous mental disorder	Z-drug abuse and dependence
3.4	Is prolonged use of Z-drugs (≥ 4 weeks) associated with the development of tolerance and dependence?	Adults with insomnia	Z-drug use ≥ 4 weeks	Z-drug use < 4 weeks or placebo	Z-drug tolerance and dependence
3.5	Is Z-drug abuse more prevalent in younger individuals (aged 18–64 years)?	General population	Adults with insomnia	Elderly individuals with insomnia	Z-drug abuse and dependence
3.6	What is the effect of a history of chronic diseases (chronic pain, neoplasms, COPD, and OSA) on Z-drug abuse?	General population	Any previous chronic clinical disorder	No previous chronic disorders	Z-drug abuse and dependence
3.7	What is the effect of different forms of zolpidem administration on dependence and abuse?	Adults with insomnia	Zolpidem in any presentation	Zolpidem in any presentation	Z-drug abuse and dependence

Abbreviations: ADHD, attention deficit hyperactivity disorder; CBT-I, cognitive behavioral therapy for insomnia; COPD, chronic obstructive pulmonary disorder; NA, not applicable; OSA, obstructive sleep apnea; PICO, Population, Intervention, Comparison, Outcome.

### Levels of evidence and critical review

The analysis of the level of evidence was conducted using the Oxford Centre for Evidence-Based Medicine (OCEBM) Levels of Evidence tool. 14 This framework assigns evidence levels from 1 to 5 based on methodological rigor, typically privileging systematic reviews and high-quality primary studies. It offers specific criteria tailored to various categories of clinical research questions, including prevalence, diagnosis, prognosis, treatment (benefits and common and rare adverse effects), and screening.

Each research question addressed in the current study was assigned to one of the OCEBM evidence categories. The specific criteria used for each relevant category are detailed in **Supplementary Material Table S2** . Using these criteria, all articles selected for each research question were individually assessed and assigned a level of evidence. This classification was performed by a single reviewer (GNP).

In addition to the level-of-evidence classification, a critical review was conducted with the aim of synthesizing conceptual, clinical, and practical aspects pertinent to each working group. Therefore, the groups received a curated selection of articles from the systematic reviews. These materials could be supplemented by additional, non-systematically identified references, based on the discretion and clinical judgment of each group. This approach ensured that each group could incorporate the most relevant and current evidence into their discussions. Ultimately, the goal was to enhance the quality of the recommendations and foster a more profound understanding of the subject matter among all participants.

### Consensus

The recommendations herein presented were developed using the Delphi method, a widely-accepted technique to achieve expert consensus through the systematic collection and analysis of opinions from a panel of specialists. 15 16 17 The Delphi method has been previously used with success, including in the development of national consensus statements by the Brazilian Sleep Association, currently called Brazilian Sleep Academy (Academia Brasileira do Sono, ABS, in Portuguese) 6 18 19 and international guidelines. 20 21

The Delphi method is characterized by four core principles: anonymous participation, iterative rounds of voting, controlled feedback between rounds, and statistical aggregation of responses. 6 22 23 In practice, it involves selecting a panel of experts who anonymously respond to structured questionnaires across multiple rounds. After each round, the participants receive a summary of the group's responses and their own, allowing for reflection and the opportunity to revise their opinions. This iterative process continues until predefined criteria for consensus are met.

The procedures adopted in the current study followed the best practice guidelines recommended by the Guidance on Conducting and Reporting Delphi Studies (CREDES) 21 and the Enhancing the Quality and Transparency of Health Research (EQUATOR) network. 24 Further methodological details, including the composition of the expert panel, are provided in **Supplementary Material Figure S1** .

### Voting process

Each working group was instructed to formulate practical items relevant to its respective topic. All proposed items were reviewed and had their wording standardized by the steering committee to ensure consistency. Items from the Z-drug dependence group were organized into two subdomains: potential for developing dependence and risk factors for dependence. Items related to deprescribing interventions were categorized into four subdomains: general considerations, non-pharmacological interventions, pharmacological interventions, and other interventions. Items related to withdrawal interventions were voted on separately for adults and for the elderly.

Additionally, all items were classified into two categories, which determined the voting methodology applied:

- **Theoretical/contextual items:** These comprised statements addressing the possibility, feasibility, accuracy, relevance, or applicability of specific aspects related to Z-drug deprescribing, abstinence, or withdrawal. This classification was applied to all items concerning dependence, withdrawal syndrome, and general considerations about withdrawal. These items were evaluated on a five-point Likert scale, with the following response options: *Totally agree* , *partially agree* ; *neither agree nor disagree* ; *partially disagree* ; and *totally disagree* .
- **Practical items:** These described specific interventions that could be implemented in the deprescribing of Z-drugs. Panelists were instructed to consider both efficacy and safety when casting their votes. These items were assessed using four response categories:
○ **Recommended:** Interventions considered as first-line options for Z-drug deprescribing, treatment of Z-drug dependence, or management of Z-drug withdrawal syndrome.
○ **Optional:** Interventions that may be used as adjuncts to recommended practices, or as alternatives when the recommended practices are ineffective.
○ **Not recommended:** Interventions that should not be used in the context of Z-drug deprescribing, dependence treatment, or withdrawal management.
○ **I don't know:** To be selected when the panelist does not feel adequately informed or confident to provide an opinion on the item.

This Delphi panel was designed to include up to three rounds of voting, with each item being eligible for a maximum of two rounds of evaluation. All rounds were conducted via an electronic form hosted on Google Forms, they remained open for one week, and voting was carried out exclusively online and asynchronously. The structure of the voting rounds was as follows:

- **Round 1:** After providing informed consent and agreeing to the authorship and participation terms, the panelists were granted access to the voting platform. The items were organized into three domains corresponding to the consensus themes and working groups: Z-drug dependence, withdrawal syndrome and side effects of Z-drugs, and Z-drug deprescribing. At the end of each domain, the participants could suggest new topics or items for inclusion in the subsequent round if they deemed them pertinent and not yet addressed. Upon completion of voting, each panelist automatically received a copy of their submitted responses.
- **Round 2:** This round included two categories: items that did not achieve consensus in round 1 and new items proposed by panelists in the previous round. For items revisited from round 1, aggregate voting results were shared to enable the panelists to reassess their responses considering the group's opinions. Only new items deemed valid and relevant by the steering committee were included in this round.
- **Round 3:** This final round included only the items that had been suggested during round 1 and failed to reach consensus in round 2.

### Consensus estimates

At the conclusion of each voting round, consensus for each item was determined. The threshold for consensus was established at 66%, meaning that an item was considered to have reached consensus if at least two-thirds of the responses were aligned. For *theoretical/contextual* items, the “strongly agree” and “partially agree” responses were grouped, as were “strongly disagree” and “partially disagree,” enabling the assessment of consensus across three response categories: *agree* , *neither agree nor disagree* , and *disagree* .

For *practical* items that failed to reach consensus during the second round of voting, the item was classified as *optional* if the combined proportion of “recommended” and “optional” responses exceeded 66% of the total votes.

All voting items and their respective results were subsequently reviewed in a joint meeting of the steering and advisory committees. The steering committee reserved the right to modify the consensus status of any item, contingent upon unanimous approval from all members of the advisory committee. Moreover, the steering committee retained the authority to exclude items deemed inapplicable or inappropriate due to political, practical, or legal considerations.

## RESULTS

### Systematic reviews – qualitative results

Each article included in the final sample was mapped to one or more of the initially-defined PICO questions ( **Supplementary Material Table S3** to **S5** ). Among the 13 articles selected for the deprescribing review ( **Supplementary Material Table S3** ), only 6 were randomized controlled trials (RCTs). Most studies allowed the inclusion of various classes of hypnotics, often grouping Z-drugs with BZDs, which limits the ability to draw specific conclusions regarding the efficacy of deprescribing strategies tailored to individual drug classes.

With respect to the types of interventions assessed, four studies examined pharmacological strategies for Z-drug withdrawal, including melatonin, ketamine, and paroxetine. The remaining articles focused on non-pharmacological interventions, such as cognitive behavioral therapy for insomnia (CBT-I), other psychotherapeutic modalities, acupuncture, educational strategies, and gradual tapering protocols.

Among the 38 articles included in the abstinence review ( **Supplementary Material Table S4** ), 30 were case reports. The predominance of case reports supports the hypothesis that the use or withdrawal of Z-drugs may be associated with withdrawal symptoms and other adverse effects. However, the absence of studies with larger samples precludes a reliable estimation of the prevalence of these outcomes.

In the dependence review ( **Supplementary Material Table S5** ), 24 articles were included, most of which provided indirect evidence supporting the potential for dependence associated with Z-drugs. Additional details on the systematic review results can be found in **Supplementary Material Figure S1** . In-depth analyses of the articles included in each review are presented in the corresponding critical review sections below.

### Levels of evidence

The highest level of evidence was found for question PICO 1.1 (effect of CBT-I on weaning from Z-drugs for insomnia), which was assigned level 2. All other questions were assigned levels of evidence from 3 to 5, demonstrating that the level of evidence is generally low. The main limiting factor for the level of evidence is the low number or absence of studies for most of the research questions. Thus, interventions for which the levels of evidence are low should not be interpreted as ineffective interventions, but as interventions for which effectiveness is uncertain due to the lack of studies. This finding reinforces the importance of clinical experience and professional practice in issuing recommendations and consensus. Other factors contributing to the low levels of evidence were the use of suboptimal experimental designs for most research questions (such as, non-RCTs in the deprescribing review and case reports in the abstinence review) and heterogeneity in the assessment of Z drugs (often assessed in conjunction with BZDs). The levels of evidence assigned to each research question are provided in Table 2 .

**Table 2:** Research questions

PICO #	OCEBM category	Level of evidence
**Z-drug deprescription group**		
1.1	Treatment - benefits	2
1.2	Treatment - benefits	5
1.3	Treatment - benefits	4
1.4	Treatment - benefits	5
1.5	Treatment - benefits	4
1.6	Treatment - benefits	3
1.7	Treatment - benefits	5
1.8	Treatment - benefits	5
1.9	Treatment - benefits	5
1.10	Treatment - benefits	5
1.11	Treatment - benefits	5
1.12	Treatment - benefits	5
1.13	Treatment - benefits	5
1.14	Treatment - benefits	5
1.15	Treatment - benefits	5
1.16	NA	NA
1.17	NA	NA
1.18	NA	NA
**Z-drug withdrawal group**		
2.1	Prevalent negative effects	4
2.2	Prevalence	3
2.3	Treatment - benefits	5
2.4	Treatment - benefits	5
**Z-drug abuse and dependence group**		
3.1	Prognosis	NA
3.2	Prognosis	4
3.3	Prognosis	NA
3.4	Prognosis	4
3.5	Prognosis	3
3.6	Prognosis	NA
3.7	Prognosis	NA

Abbreviations: NA, not applicable; OCEBM, Oxford Centre for Evidence-Based Medicine; PICO, Population, Intervention, Comparison, Outcome.

Notes: The research questions are detailed in Table 1 . Some questions do not fit exactly into the categories described by the OCEBM and were categorized by approximation. Three questions (1.16–1.18) are primarily conceptual and were not assessed using this method.

## CONSENSUS

Tables 3^4^ to 5 present the results of the voting items for each of the working groups. The descriptive results of the voting rounds can be found in **Supplementary Material Figure S1** , and detailed information about the voting (including consensus percentage, round in which consensus was reached, and change of consensus by the steering committee) can be found in the **Supplementary Material** .

**Table 3:** Items on Z-drug dependence

Subdomain	Item	Final consensus	Final result
Dependence potential	Oral zolpidem may be associated with a risk of abuse and dependence	Consensus	Agreement
	Sublingual zolpidem may be associated with a risk of abuse and dependence	Consensus	Agreement
	Extended-release zolpidem may be associated with a risk of abuse and dependence	Consensus	Agreement
	Oral spray zolpidem may be associated with a risk of abuse and dependence	Consensus	Agreement
	Orodispersible zolpidem may be associated with a risk of abuse and dependence	Consensus	Agreement
	Sublingual zolpidem has a greater potential for dependence compared to oral and extended-release formulations	Consensus	Agreement
	Zopiclone may be associated with a risk of abuse and dependence	Consensus	Agreement
	Eszopiclone may be associated with a risk of abuse and dependence	Consensus	Agreement
Risk factors	Individuals with a family history of substance abuse are at increased risk of developing substance use disorders	Consensus	Agreement
	Individuals with a history of abuse of alcohol, sedatives, or other hypnotic drug are at increased risk for Z-drug abuse	Consensus	Agreement
	Individuals with a history of psychiatric illnesses (such as anxiety, depression, ADHD, or personality disorders) are at increased risk of inappropriate use and abuse of Z-drugs	Consensus	Agreement
	Use of Z-drugs for > 4 weeks may lead to the development of tolerance and dependence	Consensus	Agreement
	Using doses higher than those recommended in the package insert increases the risk of dependence	Consensus	Agreement
	Z-drug abuse is more prevalent among younger individuals (aged 18–64 years).	Consensus	Agreement
	Easy access to prescriptions and lack of medical supervision may contribute to Z-drug abuse	Consensus	Agreement
	Chronic medical conditions—such as chronic pain, neoplasms, COPD, and obstructive sleep apnea—may contribute to the inappropriate use of Z-drugs, increasing the risk of abuse and dependence	Consensus	Agreement
	Health professionals, due to easier access, are at higher risk of inappropriate use and abuse of Z-drugs	Consensus	Agreement
	Individuals with a history of suicide attempts are at increased risk of misuse and abuse of Z-drugs	Consensus	Agreement
	Shift workers are at increased risk of Z-drug misuse and abuse	Consensus	Agreement

Abbreviations: ADHD, attention deficit hyperactivity disorder; COPD, chronic obstructive pulmonary disorder.

Notes: All items regarding Z-drug dependence were theoretical or contextual, and, thus, evaluated using a Likert scale ranging from “strongly agree” to “strongly disagree.” The “strongly agree” and “partially agree” responses were combined, as were the “strongly disagree” and “partially disagree” responses, resulting in three categories for consensus calculation: *agree* , *neither agree nor disagree* , and *disagree* . Detailed information on the voting results—including consensus percentages, the round in which consensus was achieved, and any changes made by the steering committee—is provided in Table S1 (Supplemental Material) .

**Table 4:** Items on Z-drug withdrawal syndrome and side effects

Item	Final consensus	Final results
All Z drugs are associated with withdrawal symptoms	Consensus	Agreement
The frequency of withdrawal syndrome is similar across all Z-drugs	Consensus	No agreement
Zolpidem is the Z-drug most frequently associated with withdrawal syndrome compared to other drugs in this class	Consensus	Agreement
Epileptic seizures have been reported as a symptom of zolpidem withdrawal	Consensus	Agreement
Epileptic seizures have been reported as a symptom of zolpiclone withdrawal	No consensus	Agreement
Epileptic seizures have been reported as a symptom of eszopiclone withdrawal	No consensus	Agreement
Delirium has been reported as a symptom of zolpidem withdrawal	Consensus	Agreement
Delirium has been reported as a symptom of zopiclone withdrawal	No consensus	Agreement
Delirium has been reported as a symptom of eszopiclone withdrawal	No consensus	Agreement
Anxiety symptoms are associated with zolpidem withdrawal	Consensus	Agreement
Anxiety symptoms are associated with zopiclone withdrawal	No consensus	Agreement
Anxiety symptoms are associated with eszopiclone withdrawal	No consensus	Agreement
Rebound insomnia has been reported as a symptom of zolpidem withdrawal	Consensus	Agreement
Rebound insomnia has been reported as a symptom of zopiclone withdrawal	Consensus	Agreement
Rebound insomnia has been reported as a symptom of eszopiclone withdrawal	Consensus	Agreement
Depressive symptoms are associated with zolpidem withdrawal	Consensus	Agreement
Depressive symptoms are associated with zopiclone withdrawal	No consensus	Agreement
Depressive symptoms are associated with eszopiclone withdrawal	No consensus	Agreement
Zolpidem withdrawal may be accompanied by suicidal ideation	Consensus	Agreement
Zopiclone withdrawal may be accompanied by suicidal ideation	No consensus	Agreement
Eszopiclone withdrawal may be accompanied by suicidal ideation	No consensus	Agreement
Restlessness is observed as a symptom of zolpidem withdrawal	Consensus	Agreement
Restlessness is observed as a symptom of zopiclone withdrawal	Consensus	Agreement
Restlessness is observed as a symptom of eszopiclone withdrawal	Consensus	Agreement
**Side effects associated with Z-drug use**		
Complex nocturnal behaviors are associated with zolpidem use	Consensus	Agreement
Complex nocturnal behaviors are associated with zopiclone use	Consensus	Agreement
Complex nocturnal behaviors are associated with eszopiclone use	Consensus	Agreement
Disinhibited behaviors have been associated with zolpidem use	Consensus	Agreement
Disinhibited behaviors have been associated with zopiclone use	Consensus	Agreement
Disinhibited behaviors have been associated with eszopiclone use	Consensus	Agreement
Sleep-related eating disorder has been linked to zolpidem use	Consensus	Agreement
Sleep-related eating disorder has been linked to zopiclone use	Consensus	Agreement
Sleep-related eating disorder has been linked to eszopiclone use	Consensus	Agreement
NREM parasomnias have been associated to zolpidem use	Consensus	Agreement
NREM parasomnias have been associated to zopiclone use	Consensus	Agreement
NREM parasomnias have been associated to eszopiclone use	Consensus	Agreement
Falls in the elderly have been associated with zolpidem use	Consensus	Agreement
Falls in the elderly have been associated with zopiclone use	Consensus	Agreement
Falls in the elderly have been associated with eszopiclone use	Consensus	Agreement

Abbreviation: NREM, non-rapid eye movement sleep.

Notes: All items related to withdrawal syndrome and side effects were considered theoretical or contextual in nature; therefore, they were rated on a scale from “strongly agree” to “strongly disagree.” For the purposes of consensus analysis, the “strongly agree” and “partially agree” responses were grouped together, as were the “strongly disagree” and “partially disagree” responses, resulting in three response categories: *agree* , *neither agree nor disagree* , and *disagree* . Detailed information on the voting process—including the percentage of consensus, the round in which consensus was reached, and any changes made by the steering committee—can be found in Table S1 (Supplemental Material) .

**Table 5:** Items related to Z-drug dependence

Subdomain	Item	Final consensus	Final result
General considerations	The withdrawal or weaning plan should be established at the time of prescribing a Z-drug	Consensus	Agreement
	Z-drug withdrawal can be managed by a sleep physician, regardless of their primary specialty	Consensus	Agreement
	Z-drug withdrawal should be conducted *exclusively* by a sleep physician, regardless of their primary specialty	Consensus	No agreement
	Z-drug withdrawal can be managed by a neurologist, even if they are not a sleep medicine specialist	Consensus	Agreement
	Z-drug withdrawal can be managed by a psychiatrist, even if they are not a sleep medicine specialist	Consensus	Agreement
	Z-drug withdrawal should be conducted *exclusively* by a psychiatrist or a neurologist, even if they are not sleep medicine specialists	Consensus	No agreement
	Z-drug withdrawal should be conducted *exclusively* by a psychiatrist or neurologist with specialization or board certification in sleep medicine	Consensus	No agreement
	Z-drug withdrawal can be conducted by a psychologist	Consensus	No agreement
	Mental status and psychiatric comorbidities should be assessed when planning withdrawal or weaning of Z-drugs	Consensus	Agreement
	The co-occurrence of another sleep disorder should be assessed for planning and management of zolpidem dependence in adults	Consensus	Agreement
Non-pharmacological treatment	Use of cognitive behavioral therapy for insomnia (CBT-I) in Z-drug withdrawal or weaning in adults	Consensus	Recommended
	Use of cognitive behavioral therapy for insomnia (CBT-I) in Z-drug withdrawal or weaning in older adults	Consensus	Recommended
	Use of acceptance and commitment therapy (ACT) in Z-drug withdrawal or weaning in adults	Consensus	Optional
	Use of acceptance and commitment therapy (ACT) in Z-drug withdrawal or weaning in older adults	Consensus	Optional
Pharmacological treatment in adults	Use of quetiapine in Z-drug withdrawal or weaning in adults	Consensus	Optional
	Use of other sedative antipsychotics in Z-drug withdrawal or weaning in adults	Consensus	Optional
	Use of trazodone in Z-drug withdrawal or weaning in adults	Consensus	Optional
	Use of other sedative antidepressants (such as mirtazapine, doxepine, amitriptiline, nortriptiline) in Z-drug withdrawal or weaning in adults	Consensus	Optional
	Use of antiseizure medications (such as pregabaline or gabapentine) in Z-drug withdrawal or weaning in adults	Consensus	Optional
	Use of longer-acting orexin receptor antagonists (e.g., suvorexant and lemborexant) for Z-drug withdrawal or weaning in adults	Consensus	Optional
	Use of ramelteon for Z-drug withdrawal or weaning in adults	Consensus	Optional
	Use of longer-acting benzodiazepines for Z-drug withdrawal or weaning in adults	Consensus	Recommended
	Use of short half-life benzodiazepines for Z-drug withdrawal or weaning in adults	Consensus	Not recommended
	Use of short-release melatonin for Z-drug withdrawal or weaning in adults	Consensus	Not recommended
	Use of extended-release melatonin for Z-drug withdrawal or weaning in adults	No consensus	
	Use of longer half-life Z-drugs for zolpidem withdrawal or weaning in adults	Consensus	Optional
Pharmacological treatment in older adults	Use of quetiapine in Z-drug withdrawal or weaning in older adults	Consensus	Not recommended
	Use of other sedative antipsychotics in Z-drug withdrawal or weaning in older adults	Consensus	Not recommended
	Use of trazodone in Z-drug withdrawal or weaning in adults	Consensus	Optional
	Use of other sedative antidepressants (such as mirtazapine, doxepine, amitriptiline, nortriptiline) in Z-drug withdrawal or weaning in older adults	Consensus	Optional
	Use of anti-seizure medications (such as pregabaline or gabapentine) in Z-drug withdrawal or weaning in older adults	Consensus	Optional
	Use of longer-acting orexin receptor antagonists (e.g., suvorexant and lemborexant) for Z-drug withdrawal or weaning in older adults	Consensus	Optional
	Use of ramelteon for Z-drug withdrawal or weaning in older adults	Consensus	Optional
	Use of longer-acting benzodiazepines for Z-drug withdrawal or weaning in older adults	Consensus	Optional
	Use of short half-life benzodiazepines for Z-drug withdrawal or weaning in older adults	Consensus	Not recommended
	Use of short-release melatonin for Z-drug withdrawal or weaning in older adults	Consensus	Not recommended
	Use of extended-release melatonin for Z-drug withdrawal or weaning in older adults	No consensus	
	Use of longer half-life Z-drugs for zolpidem withdrawal or weaning in older adults	No consensus	
Other treatments	Brief hospitalization for Z-drug withdrawal/weaning in severe cases in adults	Consensus	Recommended
	Brief hospitalization for Z-drug withdrawal/weaning in severe cases in older adults	Consensus	Recommended
	Mindfulness for Z-drug withdrawal/weaning in adults	Consensus	Optional
	Mindfulness for Z-drug withdrawal/weaning in older adults	Consensus	Optional

Notes: Items in the *General Considerations* subdomain were theoretical or contextual in nature; therefore, they were rated on a scale from “totally agree” to “totally disagree.” For the purposes of consensus analysis, the “totally agree” and “partially agree” responses were grouped, as were the “totally disagree” and “partially disagree” responses, resulting in three response categories: *agree* , *neither agree nor disagree* , and *disagree* . Items in the *Interventions* domain were considered practical and were rated as *recommended* , *optional* , or *not recommended* . Detailed information on voting results—including consensus percentages, the round in which consensus was achieved, and any changes introduced by the steering committee—can be found in Table S1 (Supplemental Material) .

### Z-drug use disorder

The *Diagnostic and Statistical Manual of Mental Disorders, 5th Edition, Text Revision* (DSM-5-TR) now uses the term *substance use disorder* to encompass a spectrum of severity, replacing earlier terms such as *addiction* and *misuse* . Z-drug use disorder is categorized under sedative, hypnotic, and anxiolytic use disorder. 25 *Medication misuse* includes any deviation from prescribed use—such as exceeding dosage, frequency, or duration—or using the drug without a prescription, often involving risky combinations with alcohol or other substances. 26

Pharmacovigilance data show that zolpidem is the Z-drug most frequently linked to abuse, dependence, and withdrawal. In France, 9 23,420 adverse-event reports were associated with its inappropriate use, with 40% classified as substance use disorder and 13.2% involving suicide attempts; zolpidem was the only substance implicated in 42.4% of the reports, and its fatality rate (of 20.3%) surpassed that of zopiclone and zaleplon. Additionally, real-world use of BZDs and Z-drugs often exceeds guideline-recommended durations, with up to 30% of new hypnotic users continuing treatment beyond 4 weeks. 27

Evidence 9 28 suggests that zopiclone carries a potential for dependence. In their pharmacovigilance analysis, Schifano et al. 9 identified 9,283 reports of suspected adverse reactions linked to inappropriate use, abuse, dependence, or withdrawal related to zopiclone; among these, 23.1% were classified as *substance use disorder* , with isolated use of zopiclone reported in 23.6% of the cases.

There are still relatively few studies 29 investigating substance use disorder associated with eszopiclone. This may be partly due to its more recent introduction into the market and limited availability in several countries. 30 31 32 Nevertheless, current evidence 33 34 35 36 37 does not support the development of tolerance or rebound insomnia following eszopiclone discontinuation in primary insomnia patients. As a result, eszopiclone has been proposed 32 as a potential therapeutic option in tapering protocols for zolpidem discontinuation.

### Risk factors for Z-drug use disorder

#### *Drug-related factors*

Evidence 3 9 38 suggests that pharmacodynamic factors—such as selectivity for specific GABA _A_ receptor subunits—and pharmacokinetic parameters—such as half-life and absorption rate—play a crucial role in determining the risk of dependence, withdrawal, and adverse effects associated with these agents. Previous studies 39 40 have suggested that high-dose zolpidem use may result in a loss of selectivity for specific GABA _A_ receptor subunits, thereby generating pharmacological effects more closely resembling those of short-acting BZDs. Medications with longer half-lives (such as eszopiclone) are generally associated with lower potential for dependence, whereas those with shorter half-lives (such as zolpidem and zaleplon) are more likely to promote repeated dosing and behavioral reinforcement. 3 The primary pharmacological properties associated with abuse potential are summarized in Table 6 . Immediate-release and sublingual formulations of zolpidem are characterized by rapid absorption and higher initial plasma concentrations, factors that likely enhance the risk of behavioral reinforcement and recreational use. Notably, the sublingual formulation achieves earlier and higher peak plasma levels than the immediate-release oral form, even at lower doses—a pharmacokinetic profile that may contribute to its greater abuse potential. 33 39

**Table 6:** Pharmacological properties of Z-drugs and potential for drug abuse and dependence

Characteristics	Putative mechanism	Zolpidem	Eszopiclone	Zopiclone
Rapid effect on the central nervous system*	Tendency to cause intense pleasurable effects (positive reinforcement)	IR: 1–2 h (+); ER: 1.5–2.5 hours (↓); SL: 0.5–3 hours (↑); oral spray:0.9 hour (↑); and oral solution: 0.5–3 hours (↑)	1–1.5 hours (↓)	1.5–2 hours (↓)
Dopamine release in the reward system	Higher addictive tendency	Probable (mainly higher dosage)	Possible	Probable
Shorter elimination half-life	Rapid elimination leads to frequent 'peak-and-trough' cycles of drug effects	IR: 2.5–3 hours (+); ER: 2.5–3 hours (+); SL: 2.4 hours (+); oral spray: 2.7–3 hours (+); and oral solution: 2.4 hours (+)	6–7 hours (↓)	4–5 hours (↓)
Rapid development of tolerance	Need for increasingly higher doses to obtain the same effect	+	?	+
Ability to induce euphoria or immediate symptom relief	Greater tendency towards drug abuse	+	+	+
Modulation of neural systems associated with emotional processing and anxiety	They create a psychological association of relief, which reinforces use	+	+	+

Abbreviations: +, present; ↓, lower; ↑, higher; ?, uncertain; ER, extended release; IR, immediate release; SL, sublingual.

Note: *Data refers to *time until peak blood concentration* (Cmax).

**Sources:** Tan et al. (2010); Reynolds et al. (2012); Boireau et al. (1990); and Heikkinen et al. (2009).

#### *Patient-related factors*

Risk factors for the misuse of prescription medications include environmental, interpersonal, and individual-level contributors. Environmental factors such as drug availability play a central role, while interpersonal and individual risks include urban living, lower level of schooling, permissive family environments, and untreated psychiatric or physical illnesses. 41 Younger age, white race, chronic pain, and psychiatric comorbidities are associated with general substance misuse, while older age is linked to sedative misuse. 27 42 In young adults, low level of schooling and use of alcohol, tobacco, and illicit drugs correlate with the inappropriate use of sedatives and hypnotics. 43

#### *Gender*

The prevalence of Z-drug use disorder may be higher in women, though the findings are inconsistent across studies. 9 44 45 Pharmacokinetic differences, such as lower activity of *cytochrome P450, family 3, subfamily A* ( *CYP3A* ) due to reduced free testosterone levels in women, result in higher plasma concentrations of zolpidem and increased adverse effects. Reflecting these concerns, the US Food and Drug Administration (FDA) revised zolpidem dosing recommendations in 2013, halving the prescribed doses for women. 46 47 48 49

#### *Age*

Advancing age has been associated with an increased risk of developing Z-drug use disorder. 9 44 50 This elevated risk among older adults is partly attributed to the higher prevalence of sleep and mood disorders in this population, as well as to age-related reductions in drug clearance. 28 51 52

#### *Medical and neurological conditions*

Z-drug use disorder may be associated with several clinical conditions, including:

1. **Chronic pain:** Pain, psychological distress, and insomnia frequently coexist and reinforce one another. Individuals suffering from chronic pain are at increased risk for the use—and potential misuse—of hypnotics to alleviate pain-induced insomnia. 53
2. **Cardiovascular and respiratory disorders:** Conditions such as congestive heart failure, bronchial asthma, and chronic obstructive pulmonary disease (COPD) can hinder sleep onset and maintenance. These disorders are also commonly associated with sleep-disordered breathing (such as obstructive or central sleep apnea), further impairing sleep and increasing reliance on hypnotics. 54 55
3. **Metabolic diseases:** Diabetes mellitus and hyperthyroidism may contribute to sleep disturbances through mechanisms such as nocturia, autonomic dysregulation, or association with comorbidities such as restless legs syndrome/Willis-Ekbom disease and obstructive sleep apnea. Inadequate recognition or mismanagement of these underlying causes may lead to inappropriate hypnotic use. 56 57
4. **Neurological disorders:** Neurodegenerative diseases, particularly those involving cognitive decline, such as Alzheimer's disease, are frequently accompanied by sleep disturbances. The coexistence of these disorders with psychiatric, metabolic, and cardiovascular comorbidities compounds the risk of hypnotic misuse or dependence. 58 59
5. **Prolonged hospitalization in older adults:** In elderly populations, extended hospital stays are linked to a higher risk of inappropriate use—including abuse and dependence—of hypnotics and opioids. This is especially true for patients over 75 years of age, those experiencing severe pain, and individuals undergoing polypharmacy. 50

#### *Sleep disorders*

When insomnia is not properly diagnosed and managed, and hypnotics are used beyond the recommended duration—particularly with the development of tolerance that leads to dose escalation or increased frequency of use—the risk of substance use disorder rises significantly. 45 Among adults, and particularly among women, hypnotic medications are frequently employed as the main strategy to cope with sleep difficulties. Factors associated with hypnotic use in individuals with insomnia include older age, social isolation (such as living alone), and unemployment. 60

#### *Psychiatric disorders*

Psychiatric disorders are frequently comorbid with insomnia. 61 Moreover, substance use disorders often co-occur. 25 Thus, there is an increasing likelihood of Z-drug misuse in individuals with underlying psychiatric conditions. Despite this plausible association, relatively few studies have systematically evaluated the link between Z-drug use disorder and psychiatric disorders; most available evidence consists of case reports and small observational studies.

Psychiatric comorbidities are common among individuals with Z-drug use disorder, particularly depressive, anxiety, and personality disorders. 28 62 63 Studies 28 have shown that a history of psychiatric illness, as well as concurrent use of alcohol or other substances, significantly increases the risk of Z-drug misuse. Greater severity of depressive symptoms has also been correlated 62 64 with a higher likelihood of developing Z-drug dependence.

The co-occurrence of alcohol use disorder with BZD or Z-drug use is frequently reported, with prevalence estimates ranging from 3 to 50%. Among individuals with alcohol use disorder, zolpidem and zopiclone are frequently used, alongside BZDs such as oxazepam, diazepam, and alprazolam. Studies 65 report that over 40% of individuals with alcohol use disorder concurrently use BZDs or Z-drugs, with more than 20% meeting the criteria for dependence. Zolpidem use has been linked to greater severity of substance use disorder compared to zopiclone. 65

Although data are limited, Z-drug use disorder may co-occur with other substance use disorders beyond that of alcohol; in a study 63 of 174 individuals with hypnotic use disorder, 83.5% met the criteria for dependence on at least 1 additional substance, including alcohol, opioids, cannabis, or solvents.

## Z-DRUG WITHDRAWAL

Withdrawal is defined by the DSM-5-TR as a clinical syndrome occurring after prolonged heavy substance use when blood or tissue levels decline, often prompting resumption of use to relieve symptoms. Withdrawal features are similar across sedative, hypnotic, and anxiolytic drugs due to their shared action on CNS GABA receptors. However, withdrawal symptoms arising during appropriate medical use of prescribed Z-drugs alone do not fulfill criteria for substance use disorder. 25

All Z-drugs have been associated with withdrawal symptoms; however, these appear to be more frequently reported with chronic zolpidem use. Signs and symptoms of Z-drug withdrawal are more commonly observed following abrupt discontinuation after prolonged use and at doses higher than those recommended. However, they may also occur in individuals undergoing chronic use at therapeutic doses. No epidemiological studies have been published specifically assessing the prevalence of withdrawal in Z-drug users. Table 7 summarizes the main clinical manifestations reported during Z-drug withdrawal. 9 66 67 In Z-drugs users, the occurrence of withdrawal symptoms has been associated 68 with longer duration of hypnotic use, lower level of schooling, non-medical use of hypnotics, previous failed attempts at withdrawal, and the presence of a negative impact on health.

**Table 7:** Clinical symptoms of Z-Drug withdrawal

**Vegetative symptoms** Nausea and vomiting; Abdominal pain; Sweating; Facial flushing; Myalgia, muscle tension, muscle cramps; Fatigue, loss of energy; Trembling; Palpitations and increased heart rate; Elevated blood pressure; Chest discomfort and precordial pain; Dyspnea; Lightheadedness; and Increased appetite **Neurological symptoms** Headache; Hyperacusis; Photophobia; Impairment of memory and attention; Impairment of voluntary movement control; Dystonia, motor tics; Speech impairment; and Seizures	**Psychopatological symptoms** Rebound insomnia; Nervousness and restlessness; Irritability; Anxiety; Euphoria; Psychomotor agitation; Verbal and physical aggression; Depressive symptoms; Visual hallucinations; Disorientation in time and space; Despersonalization and derealization; and Delirium

In a systematic review of Z-drug withdrawal cases, we identified 43 reports published between 1990 and 2025. Most were related to zolpidem use (93.0%), while 3 cases (7.0%) involved zopiclone ( **Supplementary Material Table S6** ). No withdrawal episodes associated with zaleplon or eszopiclone were reported. Among these cases, 53.5% (n = 23) involved female patients, with a mean age of 38.7 (standard deviation [SD]: ± 12.9) years. The median zolpidem reported dose was of 200 (range: 25–2,000) mg. The median duration of use was of 2.25 (range: 0.1–20.0) years, and the median abstinence period prior to symptom onset was of 30.0 (range: 4–168) hours. Three individuals (7.0%) were healthcare professionals. Delirium was reported in 8 cases (18.6%). Benzodiazepines were the most frequently used treatment to control withdrawal symptoms (n = 21; 51.2%).

A total of 24 epileptic seizures were described (55.8%). Most cases were associated with zolpidem use (95.8%), and 1 case involved zopiclone (4.2%). No epileptic seizures related to zaleplon or eszopiclone use were reported. The median maximum dose was of 250 (range: 85–1,500) mg. The median duration of medication use was of 3.0 (range: 0.25–11) years, and the median drug abstinence period prior to seizure onset was of 30 (range: 4–96) hours. Most of these were characterized as generalized tonic-clonic seizures (87.5%). Most patients with epileptic seizures were successfully treated with BZDs (n = 13; 56.5%).

## Z-DRUG DEPRESCRIBING

Multiple factors influence the success of withdrawing from any dependence-forming substance, including pharmacokinetics, elimination half-life, receptor binding affinity and specificity, dosage, duration of use, concomitant drug use, and the presence of medical or psychiatric comorbidities. These complexities may explain the lack of a universally-effective withdrawal protocol.

Z-drug deprescribing strategies must be individualized and developed collaboratively between physician and patient, considering medication-related variables, patient characteristics, clinical expertise, treatment goals, and the availability of family and social support systems. Prior to initiating withdrawal, patients should receive clear information about the process and potential withdrawal symptoms, along with assurances of adequate support throughout the process. 69

## INITIATION OF Z-DRUG PRESCRIPTION

To improve the likelihood of successful Z-drug deprescription, treatment initiation must be conducted appropriately and involve shared decision-making with the patient, family, and caregivers. This process should follow comprehensive counseling on available therapeutic alternatives, the pharmacological characteristics of Z-drugs, and their potential for dependence. 69

The initiation of treatment with hypnotics—whether BZDs or Z-drugs—should only occur after a definitive diagnosis has been established and all therapeutic alternatives, including non-pharmacological interventions, have been thoroughly explored and implemented. 6 The decision to prescribe any dependence-forming medication must be carefully discussed with the patient, as well as with their family members and/or caregivers, when appropriate. 69

When Z-drugs are indicated, they should be prescribed at the lowest effective dose and for the shortest duration possible. Caution is warranted for individuals at increased risk of developing Z-drug use disorder, including the following high-risk groups: 69

- Women;
- Older adults;
- Patients with chronic medical conditions, such as pain syndromes or neurodegenerative diseases;
- Patients with comorbid psychiatric disorders, such as depression and anxiety; and
- Individuals with a personal history of substance use disorders, including alcohol or prescription medications **.**

Patients using Z-drugs who are at increased risk of developing a substance use disorder should be closely monitored through regular follow-up appointments, with strict control over the quantity of the medication prescribed. It is essential to ensure that these patients do not escalate the dose without medical supervision. 69 However, within the Brazilian—public and private—healthcare system, there is no centralized monitoring of individual medical prescriptions. As a result, individuals who misuse medications can obtain prescriptions from multiple healthcare providers (a practice popularly known as *doctor shopping* ) without detection.

## DECISION ABOUT WITHDRAWING Z-DRUGS

The decision to discontinue Z-drug therapy should be considered in the following scenarios: 69 70

- No additional clinical benefit is observed with continued use;
- The underlying condition for which the medication was prescribed has resolved;
- The potential harm of the ongoing treatment outweigh its benefits;
- The patient has developed a substance use disorder; and
- The patient expresses a desire to discontinue the medication.

A patient may be considered an appropriate candidate for Z-drug tapering when the following criteria are met: 70 71

- They demonstrate understanding of the rationale for deprescribing and the potential harm associated with prolonged Z-drug use;
- They express motivation and adherence to the tapering plan;
- They have sufficient family or social support during the withdrawal process;
- They have no history of complicated withdrawal syndromes; and
- There is no clinical indication for urgent discontinuation, such as a high risk of respiratory depression, recent overdose, or evidence of Z-drug misuse.

Once the need for and the indication of Z-drug deprescription have been established, the subsequent steps should include:

- Formulating a therapeutic plan that incorporates pharmacological and non-pharmacological strategies;
- Assessing the need for inpatient care;
- Estimating the approximate duration of the withdrawal process; and
- Providing support in the event of withdrawal symptoms.

All of these steps must be explained in detail to the patient, their family, and caregivers. 69

During the deprescription period, for outpatients, it is recommended that follow-up assessments be scheduled at shorter intervals to monitor progress and evaluate the success of the withdrawal process. The likelihood of complications during Z-drug deprescription is higher among patients who have been treated with higher doses or for prolonged periods, those who have previously experienced withdrawal symptoms, subjects with a personal history of substance use disorder, or individuals who are using medications with shorter half-life. 69

Although outpatient follow-up is sufficient for most patients undergoing Z-drug tapering, certain clinical scenarios may require initiation of the withdrawal process during hospitalization. Hospital-based tapering should be considered in the presence of any of the following: 71

- History of alcohol or other substance use disorders;
- Previous episodes of drug withdrawal-related seizures;
- Presence of severe medical and/or psychiatric comorbidities;
- Use of high doses of Z-drugs; and/or
- Concurrent use of psychostimulants or opioids.

## WITHDRAWAL PROCESS

Only a limited number of RCTs have specifically investigated therapeutic options for discontinuing Z-drugs. Moreover, most existing studies group BZDs and Z-drugs, offering little differentiation between these pharmacological classes. Research on Z-drug withdrawal has predominantly focused on zolpidem, with minimal attention to other agents in the class. As a result, there is no current consensus or established gold standard for the deprescribing of Z-drugs. Given the scarcity of robust evidence specifically addressing Z-drug deprescribing and the similarities between BZD and Z-drug substance use disorder, the present review also incorporates findings from studies on BZD withdrawal.

### Non-pharmacological treatment

In addition to being recommended as the first-line treatment for chronic insomnia disorder, 5 6 CBT-I has also been investigated 72 73 74 75 76 77 78 79 as a therapeutic strategy to support hypnotic deprescription, including Z-drugs.

Individual, face-to-face CBT-I appears to be the most effective modality to support the deprescription of hypnotic agents, including BZDs 75 and Z-drugs. 73 74 However, online CBT-I, evaluated in a single study 76 involving an older adult population, did not prove effective in facilitating Z-drug discontinuation compared to the controls after 6 to 12 months of follow-up. Findings regarding self-guided CBT-I are inconsistent. While some studies 78 79 have demonstrated a significant reduction in Z-drug use among individuals in the intervention groups compared to the controls, another study 77 reported no significant effect on hypnotic discontinuation.

In a meta-analysis, Lynch et al. 72 (2020) assessed the efficacy of brief interventions in primary-care settings to reduce or discontinue the long-term use of BZDs/Z-drugs. Strategies such as instructing patients to use Z-drugs only when necessary, reducing the dose, providing positive reinforcement through reward letters upon discontinuation, brief consultations with healthcare providers, and distribution of printed educational materials, proved significantly more effective than standard care. Patients who received these interventions were more likely to discontinue BZD/Z-drug use at 6 months (risk ratio [RR] = 2.73; 95%CI = 1.84–4.06) and 12 months (RR = 3.41; 95%CI = 2.22–5.25). 72

In a meta-analysis, Soni et al. 80 (2023) assessed the effectiveness of adding non-pharmacological interventions to BZD/Z-drug withdrawal strategies. Combining non-pharmacological support with gradual dose reduction significantly improved the success of deprescribing compared to gradual tapering alone, both in the short (RR = 2.02; 95%CI = 1.41–2.89) and long terms (RR = 2.45; 95%CI = 1.56–3.85). 80

### Pharmacological treatment

Various therapeutic strategies may support the process of Z-drug withdrawal, and these should be tailored to the individual clinical context and needs of each patient. Nonetheless, the most effective method to deprescribe Z-drugs remains unclear. 70 Available alternatives include:

*Abrupt discontinuation of Z-drugs* , as the term implies, refers to the immediate cessation of the medication, without gradual tapering. This strategy may be appropriate for highly-0motivated patients who have used the medication at therapeutic doses and for a relatively short period. On the other hand, this approach is not recommended for deprescribing Z-drugs in patients who have been using supratherapeutic doses or have engaged in long-term use, given the heightened risk of severe withdrawal symptoms, including seizures and delirium. 70

*Gradual dose reduction* (GDR) refers to the progressive tapering of the medication dose to minimize withdrawal symptoms and reduce the risk of insomnia recurrence. Although protocols may vary, a commonly recommended approach involves reducing the dose by 10 to 25% every 1 to 2 weeks. More gradual discontinuation of BZDs/Z-drugs does not appear to significantly reduce the occurrence of withdrawal symptoms, as similar outcomes have been observed with tapering durations of 3 and 12 months. 70 This strategy is typically indicated for patients using therapeutic doses of zolpidem. Stabilizing the dose of Z-drugs before initiating tapering may be implemented. Some GDR protocols have adopted flexible tapering schedules that allowed for dose adjustments based on patient-reported symptoms. Patients should be informed about the risks associated with abrupt discontinuation, as well as the need to adjust the tapering rate in cases of intolerable withdrawal symptoms—slowing the reduction until the symptoms subside before resuming the taper. In some cases, short-term pharmacological support may be required to manage physical withdrawal symptoms. Regular follow-up visits and adherence to a predefined tapering schedule are essential for successful discontinuation. 81

*Substitution of BZDs* involves replacing zolpidem with an equivalent dose of a longer half-life BZD, such as diazepam or clonazepam. After substitution, the BZD dose is gradually reduced. This proposal may be indicated for patients who are using doses of zolpidem above therapeutic levels. No published studies have demonstrated that transitioning to a long-acting BZD is more effective in mitigating withdrawal symptoms than tapering Z-drugs without add-on pharmacologic interventions. 70

*Auxiliary pharmacotherapy* includes the reassessment of the patient's comorbid conditions, followed by the prescription of medications that may act synergistically in managing insomnia and the underlying comorbidity. Patients with anxiety disorders, for instance, may benefit from alpha-2-delta (α2δ) ligands or low-dose second-generation antipsychotics, such as quetiapine. In cases of mood disorders, sedative antidepressants (such as trazodone, mirtazapine, or tricyclic antidepressants) may be considered. For individuals with neuropathic pain, gabapentinoids or tricyclic antidepressants may offer additional therapeutic benefit. 70

## ADD-ON AUXILIARY PHARMACOTHERAPY

The use of pharmacological strategies involving the addition of a second agent is among the most commonly-employed approaches in the process of Z-drug deprescription. A variety of pharmacological classes have been explored for this purpose, including melatonergic agents, antidepressants (such as trazodone), antiseizure medications (such as carbamazepine, pregabalin, gabapentin), antipsychotics, BZDs, dual orexin receptor antagonists (DORAs), and even sedative agents such as ketamine. 81

## DUAL OREXIN RECEPTOR ANTAGONISTS

Ozone et al. 82 (2024) investigated the direct transition from Z-drugs to lemborexant, other DORAs, or ramelteon in adult women diagnosed with insomnia disorder. Around 95% of the patients successfully transitioned to lemborexant within 14 days. Lemborexant was associated with greater efficacy than suvorexant and ramelteon, showing fewer adverse events and greater improvements in insomnia symptoms. Direct switching to lemborexant seems to be a viable and potentially-beneficial strategy to facilitate BZD/Z-drug discontinuation in patients with insomnia disorder. 82

Tachibana et al. 83 (2024) assessed the effectiveness of novel hypnotics in facilitating the reduction or discontinuation of BZD/Z-drugs. The introduction of lemborexant, suvorexant, or ramelteon was associated with significant reductions in BZD/Z-drug equivalent doses. Dose reduction was significantly greater in patients receiving DORAs compared to those receiving ramelteon. Suvorexant showed a significantly greater reduction than lemborexant. 83

### Extended-release melatonin

Extended-release melatonin, which currently is not available in Brazil, has been investigated as an adjunctive agent in the process of deprescribing BZDs and Z-drugs. Its effectiveness remains controversial: while some studies 84 85 have reported beneficial effects, others 86 have found no advantage compared to placebo. The reported rates of successful discontinuation using 2 mg of prolonged-release melatonin ranged from 31 to 46.5%. 84 85 86 Among these, only one placebo-controlled trial 86 was conducted, which did not demonstrate significant efficacy of melatonin in facilitating withdrawal. The predictors of greater success with melatonin-assisted withdrawal included fewer anxiety symptoms, a shorter interval before the initiation of antidepressants during tapering (regardless of the specific agent), and a shorter overall duration of BZD/Z-drug use. 84

### Pregabalin and gabapentin

To date, no studies have specifically assessed the efficacy of gabapentin or pregabalin in the discontinuation of Z-drugs. Existing evidence indicates that they may offer benefits during BZD withdrawal, either by increasing the likelihood of successful discontinuation, 87 88 or by alleviating anxiety and withdrawal-related symptoms throughout the tapering process. 89

Bramness et al. 87 (2010) examined the effectiveness of pregabalin and gabapentin in facilitating BZD discontinuation. The findings showed that 15 to 29% of the patients discontinued BZD use after initiating treatment with either agent. Pregabalin demonstrated superior efficacy and tolerability compared to gabapentin, with 52% of patients achieving BZD-free status after 12 weeks. Among psychiatric patients, those treated with pregabalin reduced their BZD consumption by 48%, whereas the reduction was of only 14% in those treated with gabapentin. 87

Bobes et al. 88 (2012) assessed the efficacy and tolerability of pregabalin in facilitating BZD discontinuation among long-term users. Over a 12-week follow-up period, the overall success rate of BZD withdrawal was of 52%. The likelihood of successful discontinuation was not significantly affected by the specific BZD used or the presence of comorbid substance use disorders. 88

Hadley et al. 89 (2012) examined the effectiveness of pregabalin in assisting BZD discontinuation among 106 outpatients with generalized anxiety disorder. Following 12 weeks of treatment, there was no statistically significant difference in the rate of BZD cessation between the pregabalin and placebo groups (51.4% versus 37.0%). Nonetheless, individuals receiving pregabalin reported fewer anxiety and withdrawal-related symptoms compared to those receiving placebo. 89

### Carbamazepine

Carbamazepine was assessed 90 as an adjunctive treatment for BZD withdrawal in 40 patients. After 5 weeks of withdrawal, a higher proportion of individuals in the carbamazepine group remained BZD-free compared to the placebo group (95% versus 62%). However, by the end of the 12-week follow-up, this difference was no longer significant. Additionally, 28% of the participants required the introduction of antidepressants to manage symptoms of depression and anxiety during the follow-up. 90

### Trazodone

To date, no RCTs have assessed the use of antidepressants for the withdrawal of Z-drugs. Regarding BZD discontinuation, only one study 91 has investigated the potential role of trazodone in facilitating the process.

In an open-label clinical study, 91 trazodone (100–300 mg) was administered to 10 psychiatric inpatients with BZD use disorder during a short hospitalization period (2–4 weeks) aimed at BZD discontinuation. The use of trazodone was associated with a reduction in withdrawal symptoms and a decreased need for prolonged BZD use over a 12-month follow-up. Moreover, the patients experienced improvements in anxiety and depressive symptoms. The authors 91 suggested that antidepressants with pharmacological properties similar to trazodone may play a beneficial role in facilitating BZD discontinuation, particularly in individuals presenting with comorbid anxiety or depression.

### Ketamine

Garel et al. 92 (2023) investigated the use of intravenous ketamine infusions as an adjunct strategy to support BZD/Z-drug deprescription in 22 patients diagnosed with depression. Most participants had a history of unsuccessful attempts at discontinuing BZDs/Z-drugs, primarily due to withdrawal symptoms or worsening of underlying conditions. Following a median observation period of 12 months, 64% of the patients achieved sustained abstinence from BZD/Z-drug use. 92

### Published guidelines

Two previous guidelines 32 70 specifically addressed BZD/Z deprescribing. Although their approaches differ, several key recommendations are noteworthy:

- Integration of multicomponent CBT-I, either during the tapering process 32 70 or immediately following it; 32
- Gradual dose reduction of Z-drugs, either by decreasing the dose by 10 to 25% weekly, 32 or by 25% every 2 weeks, with a slower taper of 12.5% toward the end of the process. 70 In cases in which a 25% reduction is not feasible, a 50% reduction can be applied, including drug-free days; 32 70
- Considering substitution therapy with agents such as eszopiclone, daridorexant, or prolonged-release melatonin; 32 70 and
- In cases of symptom relapse during the tapering process, increasing the dose is not advised. Instead, maintaining the current dose for a longer period is recommended. 32 70

## DISCUSSION

Brazil and other countries have seen a significant surge in zolpidem misuse, particularly among individuals using the sublingual formulation. 93 Despite this growing concern, there is a scarcity of clinical trials and studies exploring therapeutic strategies to manage zolpidem dependence in adults and older populations. 81 Consequently, the development of the current consensus was imperative, addressing the limited familiarity and clinical experience of many healthcare providers—including specialists—on the subject, and aiming to support more precise and effective treatment approaches.

**Figure 1:**
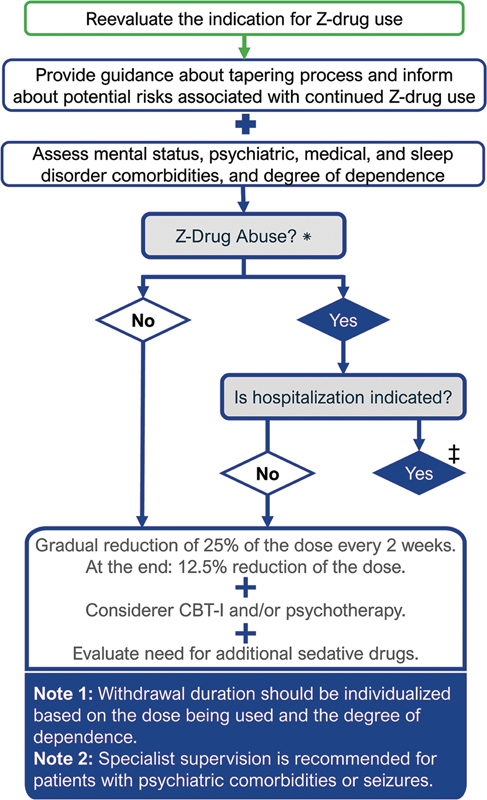
Notes: ^*^ Z-drug abuse includes any deviation from prescribed use (such as exceeding dosage, frequency, or duration) or using the drug without a prescription, often involving risky combinations with alcohol or other substances. ^‡^ The criteria to consider hospital-based tapering should include the presence of any of the following: 1) History of alcohol or other substance use disorders; 2) previous episodes of drug withdrawal-related seizures; 3) presence of severe medical and/or psychiatric comorbidities; 4) use of high doses of Z-drugs; and/or 5) concurrent use of psychostimulants or opioids. Flowchart for Z-drug deprescription.

Discontinuation strategies should be always individualized. Due to the lack of high-quality evidence, it was not possible to formulate specific recommendations for patients with complex comorbidities, such as co-occurring substance use disorders. Regarding the approaches necessary for planning withdrawal from Z-drugs, the task force recommends assessing the patient's mental state, the occurrence of psychiatric comorbidities, the severity of the dependence degree, and the co-occurrence of another sleep disorder ( Figure 1 ). In addition, this assessment aids in the choice of auxiliary pharmacotherapy and non-pharmacological treatment that may contribute to the treatment.

The task force experts recommend that withdrawal of Z-drugs should be performed by a neurologist or psychiatrist, even if they are not specialists in sleep medicine, or by sleep physicians, regardless of their primary specialty. This recommendation is in line with the curricular matrix of both specialties and the area of expertise. 94 95 96 On the other hand, the experts do not recommend that psychologists withdraw medications, since, in Brazil, these professionals do not have training and permission to prescribe and manage medications.

While the recommendation to involve specialist physicians in Z-drug deprescribing promotes greater safety and efficacy due to their specific expertise, it may also limit access to the appropriate treatment, particularly within the Brazilian Unified Health System (Sistema Único de Saúde, SUS, in Portuguese), where access to specialists is often restricted. This highlights the need for SUS administrators to implement more effective strategies to manage human resources. Furthermore, it is crucial for primary-care providers to play a proactive role in preventing Z-drug misuse by avoiding unnecessary prescriptions, identifying and addressing underlying conditions associated with a higher risk of substance use disorders, and, when dependence is identified, ensuring timely referral through regulatory systems to facilitate specialist care.

The task force holds the position that physicians are ultimately responsible for initiating treatment with medications that carry a potential for abuse and dependence. In this context, it is critical that physicians establish an accurate diagnosis, clearly define the therapeutic goals, and determine the appropriate duration of treatment. Patients, along with their family members and caregivers, should be actively involved in the decision-making process after being fully informed of the risks associated with the use of Z-drugs. The physician is also responsible for controlling prescriptions and limiting the quantity of tablets dispensed, with all such actions appropriately documented in the medical record.

### Non-pharmacological treatment

Regarding non-pharmacological therapeutic strategies, the use of CBT-I was *recommended* for both adults and older adults during the process of Z-drug deprescribing. The use of acceptance and commitment therapy (ACT) received a weaker recommendation and was classified as *optional* . The expert panel maintained the recommendation of CBT-I for Z-drug withdrawal in the elderly, based on clinical experience. There are no published studies on the use of ACT specifically for Z-drug withdrawal; however, its established role in the treatment of insomnia supports its inclusion as a potential adjunctive option during the discontinuation process. Mindfulness was also included and is considered *optional* for Z-drug deprescribing. Regarding other non-pharmacological interventions, the task force considers brief intervention for adults and the elderly a consensus.

It is important to emphasize that chronotherapeutic interventions (such as light therapy and regular physical exercise) may provide valuable adjunctive support. These strategies are recommended as complementary treatments to CBT-I and have shown benefits in improving sleep quality and reducing reliance on hypnotic medications. When integrated with CBT-I, chronotherapeutic interventions offer a comprehensive, multidimensional approach that can enhance the success of gradual tapering and discontinuation of hypnotics in patients with chronic insomnia.

### Pharmacological treatment

Regarding the pharmacological approaches to manage Z-drug (particularly zolpidem) withdrawal, a review on the feasibility of BZD/Z-drug deprescribing highlighted the potential benefits of gradual tapering combined with non-pharmacological interventions; however, the overall quality of the supporting evidence was low. 30 73 80

Pharmacological interventions involving the addition of a second agent are among the most frequently employed strategies for the deprescription of Z-drugs. Although there is limited clinical evidence 84 85 86 90 91 97 to confirm the efficacy of these approaches, expert opinion supports their use (particularly when guided by a comprehensive evaluation of patient comorbidities) as a means to facilitate Z-drug withdrawal.

### Pharmacological treatment in younger adults

The task force reached a consensus supporting the *optional* use of quetiapine or other sedative antipsychotics, trazodone or other sedative antidepressants, α2δ ligands, and ramelteon as add-on therapies in the management of Z-drug withdrawal in younger adults. However, no consensus was achieved regarding the use of immediate-release melatonin, reflecting the current lack of high-quality controlled trials assessing its efficacy. The use of intermediate- or long-acting BZDs was *recommended* , whereas short- and ultra-short-acting BZDs, as well as immediate-release melatonin, were *not recommended* . Notably, extended-release melatonin and DORAs were not evaluated in these recommendations due to their unavailability in the Brazilian pharmaceutical market. The available data support the potential usefulness of DORAs in facilitating deprescription, underscoring the desirability of incorporating this pharmacological class into the Brazilian market to assist in Z-drug withdrawal.

### Pharmacological treatment in the elderly population

In the elderly population, the level of consensus among task force members was lower, and no recommendation was made for the use of BZDs, antipsychotics, or melatonin (immediate or prolonged-release) during Z-drug deprescription. For the other pharmacological agents recommended for adults, the use in elderly patients was categorized as *optional* . The high incidence of adverse effects associated with BZDs in older adults, such as cognitive impairment, falls, and excessive sedation, supports this cautious approach. Similarly, antipsychotics are associated with increased cardiovascular risk in the elderly. 98 Additionally, slower drug metabolism and a higher prevalence of comorbidities in this population complicate the use of add-on pharmacotherapy or substitution with long-acting BZDs. Therefore, any optional pharmacological intervention in elderly patients should be individualized, with careful consideration of comorbidities, close clinical monitoring, and gradual dose titration.

### Limitations and strengths

The current consensus presents several limitations, including the lack of high-level evidence to support expert opinions, and the potential for biases stemming from the diverse clinical practices of the participating professionals. Additionally, many of the experts had limited or no experience with medications not yet available in Brazil, such as DORAs and extended-release melatonin, which may have influenced the strength of the recommendations for these agents. Another significant limitation was the failure to evaluate all psychotherapeutic approaches to manage substance dependence, including variations of brief interventions. Furthermore, the absence of psychologists with expertise in these therapeutic modalities may have restricted the comprehensiveness of the non-pharmacological recommendations.

On the other hand, these recommendations benefited from the participation of neurologists specializing in sleep medicine from all regions of Brazil, working in public and private healthcare settings. This diversity enabled the incorporation of experiences with Z-drug tapering in populations with varied genetic, socioeconomic, and cultural backgrounds. Moreover, the collaboration of a highly-experienced group of psychiatrists specializing in substance use disorders contributed valuable insights, particularly in managing cases involving severe dependence, which may arise with Z-drugs. The methodological integration of literature review and expert consensus resulted in recommendations that are aligned with European and Canadian guidelines.

In conclusion, we propose a strategic plan that begins with comprehensive medical and mental health assessments, followed by an evaluation of addiction severity, to be conducted by specialists, as thorough planning is essential for any therapeutic program. Finally, we present recommendations on non-pharmacological and pharmacological strategies, grounded in the limited available clinical evidence and in the clinical experience of specialists regarding the weaning and withdrawal of these medications.

The task force makes the following recommendations regarding Z-drug deprescription:

- Conduct a thorough assessment of the patient's mental status, psychiatric and sleep-related comorbidities, and the degree of pharmacological dependence;
- The deprescription process should be managed by a neurologist, a psychiatrist, or a specialist in sleep medicine;
- It should not be conducted by a psychologist acting independently;
- Gradual tapering of the Z-drug is advised;
- Non-pharmacological strategies are encouraged, with:
○ CBT-I being *recommended* ; and
○ ACT being *optional* ;
- In specific cases of zolpidem withdrawal, the *optional* adjunctive use of a second agent may be considered, such as:
  ○ Trazodone or other antidepressants;
  ○ Quetiapine or other antipsychotics; and
  ○ α2δ ligand agents (such as gabapentin or pregabalin); and
  ○ Alternative hypnotics (such as ramelteon, zopiclone, or eszopiclone);
- The use of intermediate- or long-acting BZDs is *recommended* ;
- The use of short- or ultra-short-acting BZDs is *not recommended* ;
- The use of immediate-release melatonin is *not recommended* ; and
- Prolonged-release melatonin and DORAs, although recommended in international guidelines, were *not* evaluated due to their unavailability in the Brazilian market.
